# Supplementary material for: The genome sequence of the Alder Moth, Acronicta alni (Linnaeus, 1767)
Source: Wellcome Open Res. 2025 Feb 12;10:68. [Version 1] doi: 10.12688/wellcomeopenres.23704.1 (PMC11885909; doi:10.12688/wellcomeopenres.23704.1)
Supplement: Peer review history

# Reviewer report: Reviewer response for version 1

**DOI:** 10.21956/wellcomeopenres.26152.r118734

**Published:** 2025-03-06

**Authors:** Sivasankaran Kuppusamy

**Affiliations:** 1 Loyola College, Chennai, Tamil Nadu, India

The chromosome-level genome assembly of Alder moth, *Acronicta alni* (Linnaeus, 1767) was done by the authors. They scaffolded 31 chromosomes from the genome sequence. This comprehensive date will be useful for the genomic analysis of the moths.

Minor comments on the manuscript

 In the background second paragraph “through the centre of the forewing around the kidney mark”. the kidney mark can be written as a reniform. 

                “through the centre of the forewing around the reniform”

 Throughout the text, the authors have used the genus full name, *Acronicta alni*. If possible, to use the full genus name at first, and later to shorten it to *A. alni*.

 In the Genome sequence report first paragraph the first sentence can be written as “The genome of *A. alni* (Figure 1) was sequenced using…………………..

 Authors have sequenced the genome’s total length of 160.15 Gb using three different sequencing platforms. After the assembly they received 470.37 Mb in size. But authors haven’t observed the protein-coding genes, non-coding genes and gene transcripts.  Give reason?

 Above all, I confirm that the manuscript meets the necessary scientific standard and is suitable for indexing.

Are sufficient details of methods and materials provided to allow replication by others?

Yes

Is the rationale for creating the dataset(s) clearly described?

Yes

Are the datasets clearly presented in a useable and accessible format?

Yes

Are the protocols appropriate and is the work technically sound?

Yes

Reviewer Expertise:

Mitogenome analysis of Noctuoidea moths

I confirm that I have read this submission and believe that I have an appropriate level of expertise to confirm that it is of an acceptable scientific standard.

# Reviewer report: Reviewer response for version 1

**DOI:** 10.21956/wellcomeopenres.26152.r118728

**Published:** 2025-02-19

**Authors:** Christophe Klopp

**Affiliations:** 1 INRAE, Castanet-Tolosan, France

The authors present the first assemblies of the Alder Moth, Acronicta alni.

 The methods are standard, the results are of high quality.

 It is unclear how hap2 was scaffolded. This should be explained.

 It is also strange not to have the same chromosome order and orientation for both haplotypes. This would ease their integration in a pangenome, for example. 

 The material and methods look a bit like a cut and paste of a previous article in which primary and alternate assemblies were produced. "primary contigs" should be replaced by "haplotype 1 contigs".

Are sufficient details of methods and materials provided to allow replication by others?

Yes

Is the rationale for creating the dataset(s) clearly described?

Yes

Are the datasets clearly presented in a useable and accessible format?

Yes

Are the protocols appropriate and is the work technically sound?

Yes

Reviewer Expertise:

bioinformatics, genome assembly and annotation

I confirm that I have read this submission and believe that I have an appropriate level of expertise to confirm that it is of an acceptable scientific standard.
